# Biogenic Metal Nanoparticles: A New Approach to Detect Life on Mars?

**DOI:** 10.3390/life10030028

**Published:** 2020-03-20

**Authors:** Marta Filipa Simões, Cristiane Angélica Ottoni, André Antunes

**Affiliations:** 1 State Key Laboratory of Lunar and Planetary Sciences, Macau University of Science and Technology, Avenida Wai Long, Taipa, Macau SAR, Hong Kong, China; 2 Bioscience Institute, São Paulo State University (UNESP), São Vicente, SP 11380-972, Brazil

**Keywords:** metal nanoparticles, biogenic synthesis, biosignatures, life detection, astrobiology

## Abstract

Metal nanoparticles (MNPs) have been extensively studied. They can be produced via different methods (physical, chemical, or biogenic), but biogenic synthesis has become more relevant, mainly for being referred by many as eco-friendly and more advantageous than others. Biogenic MNPs have been largely used in a wide variety of applications, from industry, to agriculture, to health sectors, among others. Even though they are increasingly researched and used, there is still space for exploring further applications and increasing their functionality and our understanding of their synthesis process. Here, we provide an overview of MNPs and biogenic MNPs, and we analyze the potential application of their formation process to astrobiology and the detection of life on Mars and other worlds. According to current knowledge, we suggest that they can be used as potential biosignatures in extra-terrestrial samples. We present the advantages and disadvantages of this approach, suggest further research, and propose its potential use for the search for life in future space exploration.

## 1. Introduction

### 1.1. Metal Nanoparticles (MNPs)

Nanoparticles (NPs), particles within the nanometer range, can be very different and have a huge scope of applications [1,2]. Metal nanoparticles (MNPs) are inorganic particles that can be constituted by noble metals (e.g., gold, silver, and platinum), magnetic metals (e.g., iron, cobalt, and nickel), or semiconductors (e.g., oxides of titanium, zinc, and cadmium) [1].

MNPs have unique physical properties, high surface-area-to-volume ratio, and are clearly distinguished from bulk metals due to their reactivity, as well as their mechanical, electromagnetic, chemical, and optical properties, which are conferred by the confinement effect [3,4,5]. MNPs often display surface Plasmon resonance, which leads to absorption in the UV–Vis region and distinct optoelectronic characteristics, which facilitates their detection. However, different sizes and shapes will have different absorption spectra and interparticle properties [6].

**Figure 1:**
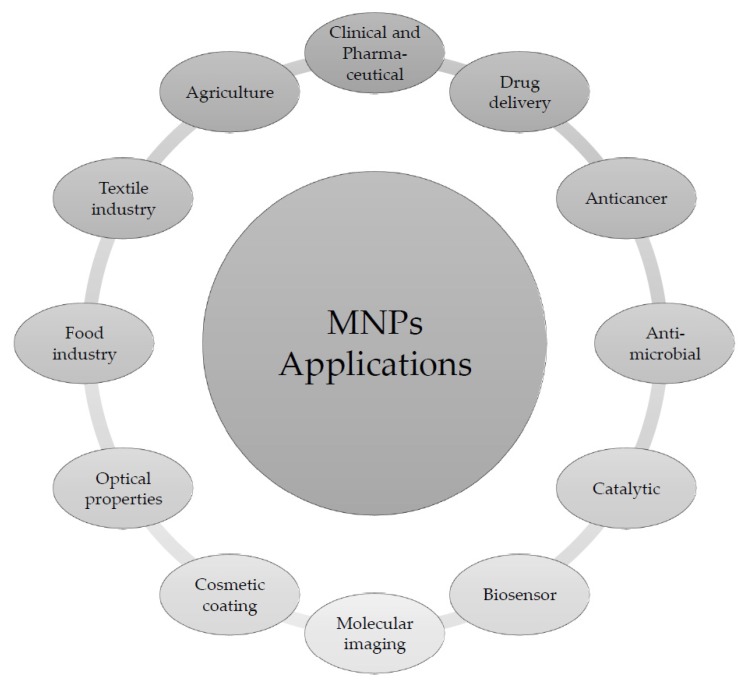
Applications of Metal nanoparticles (MNPs; adapted from [2,4,5,7]).

With the increased development of nanotechnology and its research, many applications of MNPs have already been implemented (Figure 1) and more developments are expected [4].

MNPs are very advantageous in microbial detection and biosensing, both in clinical and food sectors, because of their properties: a large surface area that enhances biorecognizers and receptor immobilization, good ability for reaction catalysis and electron transfer, and good biocompatibility. Moreover, MNPs can be used on their own or combined with other types of molecules, structures, and nanostructures. MNP-based sensors can lead to significant signal amplification, higher sensitivity, and great improvements in the detection and quantification of biomolecules and different ions [8].

### 1.2. Different Types of MNPs

MNPs can have different metals on their compositions, but the most researched and used are gold and silver NPs.

#### 1.2.1. Gold NPs (AuNPs)

Gold NPs (AuNPs) have a wide range of applications. They are biocompatible, easily interacting with biomolecules (e.g., antibodies, nucleic acid probes, and glycoproteins) and thus considered safe for in vivo and clinical applications. Regarding microbial detection, AuNPs are one of the preferred choices due to their surface Plasmon band localization in the visible spectrum, ease of synthesis, various functionalization, ability to quench fluorescence, excellent biocompatibility, and low toxicity [9,10].

The food industry in particular has developed a very wide range of uses for AuNPs. These NPs have been applied as sensors, e.g., to assess Ca^2+^ concentration in meat or to detect mycotoxins in food, and biosensors to detect microbial contaminants. Another application is their use for removal of contaminants in food, e.g., bisphenol A (BPA) from milk [7].

#### 1.2.2. Silver NPs (AgNPs)

The antimicrobial properties of silver have been known for a very long time and used extensively throughout human history. However, the application of these properties has been massively boosted since it started being engineered into MNPs [3,11].

The popularity of silver NPs (AgNPs) is due to their localized surface Plasmon resonance, good conductivity, chemical stability, catalytic and broad-spectrum anti-microbial activities, as well as cytotoxic effect on cancer cells [11]. They have been employed mostly for their antimicrobial capacity in the medical, food packaging and textile (by incorporation in hybrid materials), and agriculture industries, but also for catalytic, anticancer and antioxidant applications, antiviral action and anti-inflammatory activity (e.g., for HIV), in cosmetics, electronics, and energy [5,7,11,12,13]. Besides their activity against microorganisms, they also prevent microbial growth and inhibit the formation of biofilms, making them very useful as a component of many materials and tools for the food industry, biomedical applications, engineering sciences, and agriculture [7].

Smaller sizes of AgNPs are preferred for many applications, namely, for their antimicrobial activity. Smaller AgNPs have a greater tendency to enter into organisms, a larger number of surface atoms available for diverse reactions, higher release of Ag ions, and increased production of reactive oxygen species (ROS) on their surface, which leads to an increased toxicity [12].

#### 1.2.3. Other MNPs

Copper NPs (CuNPs), cobalt NPs (CoNPs), and MNPs with other alternative metals have also been explored. These include palladium NPs (PdNPs), selenium NPs (SeNPs, using sodium selenite solution as the precursor for reduction), platinum NPs (PtNPs, using a platinum chloride, PtCl_4_, as precursor), and lead NPs (PbNPs) [14].

The synthesis of CuNPs, which uses copper acetate as the common precursor, is seen as more cost-effective than AgNPs, AuNPs, and PtNPs [14,15].

CoNPs have been used for their antimicrobial capacity, e.g., against *Acanthamoeba castellanii*, a facultative pathogen that can cause several diseases, some with high associated rates of mortality and morbidity, and for which there is no known drug available [16].

Magnetic NPs are another type with application in diverse formats: fluorescent magnetic NPs, antibiotic- and other organic compound-modified NPs, in microarrays, as amino-functionalized silica-coated NPs in combination with polymerase chain reaction (PCR), NPs conjugated with antibodies, and as quaternized NP fluorescent polymer. Many of these are used to label bacteria for posterior detection with techniques such as flow cytometry and flow cytometric determination of magnetic nanoparticle collection and separation [17].

### 1.3. MNPs for Specific Microbial Detection

Microbes can be difficult to detect and identify, especially when they are slow-growing, present in low densities, or produce insignificant amounts of antibodies to allow their early detection. Microbial identification and detection rely on several different methods, but most of the classic approaches are too slow and not sensitive enough.

The last decades have seen great development in alternative nanotechnology-based methodologies (Table 1), and many of them make use of MNPs (e.g., in diagnostics to detect pathogens, their products, and antibodies produced in response to their presence).

The major functions of MNPs for microbial detection are immobilizing bioreceptors, mediating electron transfer, catalyzing bioreactions, amplifying mass change, and enhancing refractive index changes [8]. Nevertheless, these applications do not necessarily distinguish between viable and non-viable microorganisms. However, these MNP-methods are simple, rapid, reliable, sensitive, and early-screening methods, and preferable to conventional procedures such as the ones that involve microbial cultures and growth, which are lengthy and sometimes difficult to perform. These techniques have all been developed and used for detecting only target species or groups and signs of their presence. In all of them, an interaction is developed between the nanostructure and the microorganism [17]. Even though all these methods have been used to detect microbial life, they are not applicable as biosignatures directly and, they cannot be adapted for this purpose.

### 1.4. Synthesis of MNPs

There are two major approaches to the synthesis of MNPs:

(1) “top-down” approach, which involves restructuring a bulk material in order to create a nanomaterial. This is not the best-suited method to achieve very-small sized particles [2].

(2) “bottom-up” approach, which implies the construction of a nanomaterial from basic building blocks. It involves the formation of the nanostructures through self-assembly, atom-by-atom, molecule-by-molecule, or cluster-by-cluster [1]. This approach illustrates the possibility of creating materials that are designed to have exactly the desired properties (atomic level) [2].

#### 1.4.1. Physical Synthesis of MNPs

The physical methods for the synthesis of MNPs are “top-down” approaches, based on the subdivision of bulk metals. These include mechanical crushing, pulverization of bulk metal, and discharge between metal electrodes. MNPs produced by these methods are larger-sized and have wider size distribution when compared to other NPs [2]. The NPs obtained through physical synthesis include the vaporization of metals and their deposition and combination onto substrates, leading to the formation of nanosized structures [20].

There are several methods of physical synthesis: (1) for vapor phase: chemical vapor condensation, gas evaporation, arc discharge, hydrogen plasma, laser pyrolysis; (2) for liquid phase: microemulsion, hydrothermal, solgel, sonochemical, and decomposition processes; and, (3) for solid phase: ball milling (which involves crushing of metal microparticles) and mechanochemical synthesis [2,21].

#### 1.4.2. Chemical Synthesis of MNPs

Chemical synthesis of MNPs implies the reduction of metal salts/ions in solution or decomposition of precursors, to form small assemblages of metal atoms, via the use of hazardous chemicals such as sodium borohydride, tetrakishydroxymethylphosphonium chloride (THPC), poly-N-vinyl pyrrolidone (PVP), and hydroxylamine [20], as well as solvents like sodium dodecyl sulfate (SDS), sodium borohydrate, hyperbranched polyester, and hydrocarbons [22], followed by aggregation of atoms [2]. Some of those chemicals are also used to stabilize the NPs [13].

Besides the chemical reduction, other “bottom-up” methods used for MNPs formation are pyrolysis and the sol-gel method [22].

Thermolysis, another chemical method, occurs with dissociation of organometallic precursors at high temperatures with the use of organic solvents. Sometimes, surfactant is added to the reaction medium to reduce the coalescence of particles [21].

#### 1.4.3. Biological Synthesis of MNPs

Biological synthesis of MNPs usually follows the “bottom-up” synthesis approach [1]. Biological systems (e.g., plants and microorganisms) display a vast variety of biomolecules with reductive properties. Briefly, a liquid solution containing reducing and stabilizing agents is used; atoms are formed from the reduction of a metal ion (e.g., Ag^+^), which agglomerate into oligomeric clusters and eventually form MNPs (e.g., AgNPs) [11]. Note that the full mechanism of the reactions responsible for the biosynthesis processes are not yet fully understood, and many details remain unclear [6]. Most studies on MNPs are unfortunately focused exclusively on the final products and applications rather than on the understanding of these mechanisms.

The capacity for biosynthesis of MNPs has a remarkably widespread distribution (Table 2), including representatives of Bacteria, Archaea, and Eukarya and even extends to viruses [13,20,23]. In general screenings for testing production, some species and strains are classified as non-producers (e.g., [5,24,25,26]). This is likely a misclassification that reflects either low yields or the unsuitability of tested conditions to promote MNPs production by these species. Despite the lack of systematic testing of different taxa, MNPs production seems to be universal to all living organisms rather than restricted to specific groups. The table below illustrates the diversity of these organisms and their derived molecules that can be used for MNP production.

Microorganisms are constantly exposed to metals and often have inherent defense reductive mechanisms that mediate the synthesis of a variety of MNPs [1]. Many microorganisms are capable of synthesizing inorganic-based materials [39].

Biogenic processes can be affected by pH, temperature, time of reaction, the concentration of precursor, culture media, the quantity of biomass, agitation, the quantity of microbial biomass, and light intensity. All these parameters can influence the final MNPs formed (shape, size, concentration) and their rate of formation; they can be optimized for specific results with variation from species to species [5,6,13,30]. Filamentous fungi are the best-studied organisms for this biosynthesis, and conditions for mycogenic synthesis of MNPs vary as well as the resulting NPs, but according to Zhao et al. [11], for AgNPs, the best conditions are pH 7, at 25 °C with 1 mM silver nitrate (AgNO_3_), and 15-20 g of wet fungal-cell filtrate. Also, Guilger-Casagrande and de Lima [13] put together a list of optimized conditions for mycogenic AgNPs production that varies for each different species and strain.

#### 1.4.4. Comparative Overview of MNPs Synthesis Processes

The synthesis of nanoparticles by non-biogenic methods requires an additional step: functionalization. In it, the MNPs surfaces are coated using polymers and surfactants. In biogenic processes, the capping of NPs occurs at the same time that NPs are formed, using biomolecules from the organism used in the synthesis process. Hence, no additional steps are required. The biomolecules, which originate from the reducing organism, are highly capable of binding to metal, forming cappings that contribute to more stable NPs and prevent their agglomeration and aggregation [13]. The differences between processes lead to a combination of different advantages and disadvantages for each one (Table 3).

### 1.5. Astrobiology and the Search for Life on Mars

Astrobiology is a cross-disciplinary research field combining approaches from biology, geology, chemistry, and planetary sciences to study the origin and development of life in the universe and search for extinct and currently existing organisms in other worlds. Mars, which is the most Earth-like planet in the Solar System, is currently the main target for the majority of astrobiological studies.

The existence of water is seen as essential for life’s existence and subsistence, so this has been a central point of discussion on the exploration of the Red Planet. Despite the currently barren Martian landscape, there is significant evidence of a past of abundant liquid water, particularly pronounced in the Noachian around 4.1–3.7 Gya ago (e.g., [40,41]). Current conditions on the surface of Mars (low temperature and pressure) drastically reduce the stability of liquid water, which would quickly sublimate or freeze, but high concentrations of salts could stabilize localized temporary small liquid brine pools.

The presence of relatively abundant perchlorate salts in Gale Crater (detected by the Curiosity Rover mission) and their widespread distribution on Martian soils would be conducive to such effects [42]. Perchlorates could further contribute to this as they can form stable, hydrated compounds and liquid solutions by deliquescence and absorption of atmospheric water vapor. Seasonal changes and spectral evidence in recurring slope *lineae* on Mars suggest the existence of current brine flows or seeps associated with perchlorates and its hydrated salts [43]. Given the more sheltered conditions in the subsoil, larger-scale sub-surface liquid brine (and/or water deposits) are likely and could be the source of these brine outflows (e.g., [44]).

The current surface of Mars is exposed to multiple extremes and seen as inhospitable and uninhabitable [45]. These include low temperatures, low pressure, very dry and oxidized conditions, and high levels of ultraviolet (UV) and ionizing radiation exposure, all of which are due to the very sparse Martian atmosphere [45,46].

### 1.6. Biosignatures

When searching for extraterrestrial life on Mars and elsewhere, it is of uttermost importance to define what constitutes a biosignature (evidence of biological processes), and how it relates and differs from “abiosignatures”/abiotic features (derived from non-living ones), particularly to avoid misidentification or wrongful detection [46,47]. One should note that life evades definition and is remarkably complex from a conceptual perspective, which might hamper a clear-cut separation between biotic and abiotic. In addition to this issue, we bias our analysis by grounding it on the single example provided by our own planet and its life-forms. Despite such limitations, it is based on such Earth-centric studies that we now know that traces of life can be detected on the crust of our planet and that life is present in many extreme locations previously considered sterile [48]. The study of such extreme environments, particularly those defined as terrestrial analogs, provide us with vital information for astrobiological exploration of other planets and moons [48,49,50].

Any detectable sign, matter, gas, or pattern that strongly suggests a biological origin and can thus be used as evidence indicating the existence, past or present, of living organisms is a biosignature [48,51]. By definition, they are objective, quantifiable characteristics of biological processes. They can be atmospheric biosignatures if referring to the presence of one or several gas species, surface biosignatures if detectable by their light reflection characteristics which allows them to be detected by remote sensing, or bioindicators if they suggest a biological origin but can also be produced abiotically [51]. Biosignatures can be divided into three different categories (Table 4): substances, objects, and patterns [48].

When it comes to looking for life on Mars, it has to be considered that early Earth had anaerobic environments similar to the ones on early Mars, which lead to the studies of microfossils and their biogenicity and syngenicity. However, sample preparations and complex methodologies and equipment do not facilitate the search for fossil microorganisms [45]. Therefore, all possible alternatives are of interest.

Signatures of any biological activity can be detected using a wide range of options and their detection is relevant for several fields. As an example, the use of biomarkers and their detection have been largely used in medical science for disease diagnostics. In such clinical settings, nanotechnology is increasingly employed for early identification of viruses and pathogenic bacteria through rapid and direct detection of biomarkers (e.g., nanowires, nanotubes, nanoparticles, cantilevers, microarrays, and nanoarrays) [17].

## 2. Hypothesis and Discussion

If life ever existed or still exists on Mars, it was or is probably microbial, free-living in fluids or as biofilms in sediments, and probably with carbon-based origins developed in the presence of water, similar to the first life forms on Earth [52]. Such microbial biomass, regardless of their physiological characteristics, might still be preserved in Martian sedimentary rocks or fluids [46,53], or any biological molecules might be trapped in mineralized cells [47]. It is known that the subsurface of Mars has mostly acted as a giant freezer, potentially preserving any remains of Martian life [54]. Under these assumptions, we propose a method for preliminary screening of life that presents several advantages: it is simple, relatively fast, cheap, without the need of advanced, specialized, and dedicated equipment and personnel, and easy to perform and implement either on Earth or, with some adaptations, in situ. In this regard, prior experiences of adapting detection techniques and inserting them in chip-based systems (e.g., the Life detector LDChip [55]) are expected to be most beneficial.

### 2.1. Proposed Method

**Figure 2:**
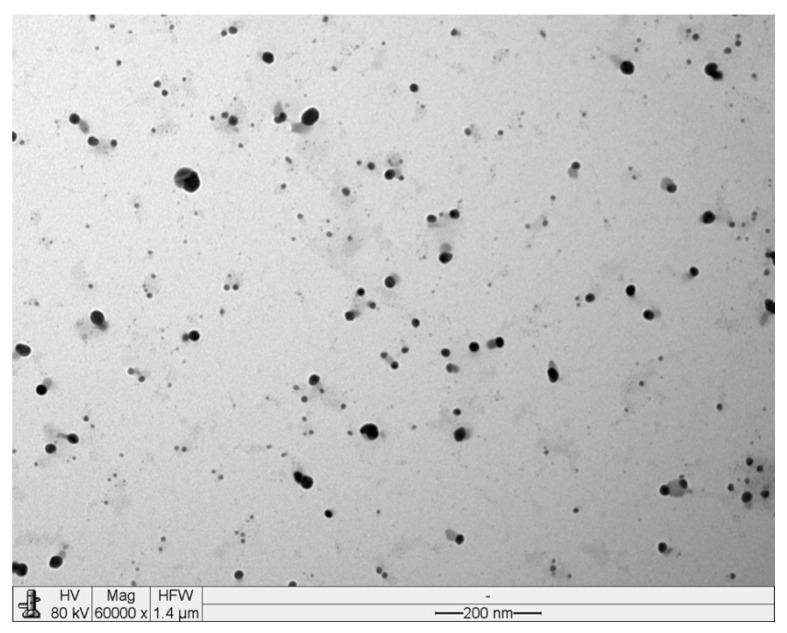
Transmission electron microscope (TEM) micrograph of a MNP suspension, with mycogenic silver NPs (black dots) synthesized by an *Aspergillus niger* strain. Scale: 200 nm.

For any sample collected from Mars, we propose a bottom-up process involving several steps. Taking into consideration the most studied and used biosynthesized MNPs (AuNPs and AgNPs), and adapting from the standard biological synthesis methods, based on microbial synthesis mostly done with axenic cultures, we propose the following methodology:

(A) Retrieve an amount of sample with a known mass/volume. Dilute it, if soluble, in a determined quantity of water. If the sample is not soluble in water, then directly use a defined volume of sample (if liquid/aqueous). Solid samples should be smashed or crushed, whenever possible, then vortexed with a defined amount of water, in order to dilute it or wash it, for several minutes.
(B) Once mixed with the water or directly aliquoted, filter the sample first with a filter paper, to remove larger fragments of material, and then with a 0.1 or 0.22 µm filter in order to remove any possible biomass and achieve a sterile solution.
(C) Treat this solution with a precursor: AgNO_3_ (final concentration of 0.1–5 mM) for AgNPs, or tetrachloroauric acid trihydrate (AuCl_4_·3H_2_O) (final concentration of 0.2–4 mM) for AuNPs. Allow for the reaction to happen by incubating the mixture at room temperature at 100 rpm, as you would for standard biosynthesis of MNPs. Leave for an incubation period of at least 3–5 days.
(D) During the incubation period, follow any color changes (derived from a shift in the surface Plasmon resonance of the metal ions after the reduction [56]) which will be noticeable by direct observation or by spectrophotometry (at 520 nm for AuNPs and 380 nm for AgNPs [3]). It is expected that any AuNPs solution will look red-colored, and any AgNPs will be yellow/brown-colored [3]. Note that the wave-lengths [39], color, and color intensity might vary due to the NPs’ size, uniformity, shape, dispersion, and their dielectric constant of the surrounding medium [3]. After the incubation period, if you do not detect any color change, concentrating the solution by reducing its volume through evaporation will make any changes more noticeable.
(E) Furthermore, analyze the final solution by electronic microscopy for visual confirmation of NP formation (e.g., Figure 2).

If MNPs are formed, this will mean that in the initial solution retrieved from the sample, there are likely biological substances that allow for the chemical reaction to happen and form the NPs. This will then work as an initial screening.

Further characterization of the MNPs formed can then be done by techniques such as: Fourier transform infrared spectroscopy (FTIR), for chemical identification, especially of any capping agents; X-ray diffraction (XRD), to determine the MNP’s crystal structure; energy dispersive X-ray analysis (EDAX), to determine elemental composition; dynamic light scattering (DLS), to analyze surface charge, size distribution and general quality; nanoparticle tracking analysis (NTA); time of flight-secondary ion mass spectroscopy (TOF-SIMS), to do a primary analysis of MNPs surface; low energy ion scattering (LEIS), to identify elements present in the outer surface of the MNPs; surface-enhanced Raman scattering (SERS), to detect single molecular attachments to the surface of MNPs [2].

### 2.2. Advantages and Challenges

The proposed method intends to simplify the search for life by looking for the formation of MNPs instead of searching for interactions between organisms and MNPs, as is currently the case in microbial detection using MNPs and biosensing. The advantages of this strategy are that this allows for an easy and fast screening for the existence of either past or current life on Mars and elsewhere. It is also a simple method that does not need complex and expensive equipment. It can easily be adapted to the location where life’s existence will be researched.

The use of MNPs as biosignatures for detection of life as we propose it still has some challenges:

(1) There are on-going knowledge gaps on biogenic production of MNPs: the detailed mechanistic of MNPs formation is not completely understood, optimization and control of parameters in production are not clearly defined and present too much variability.
(2) The detection of MNPs, by direct observation, color change, and spectrophotometry analysis, has limits that might not allow for detection and may give false-negative results if a very small amount of MNPs is synthesized.
(3) A positive result and MNPs formation still need confirmation that the substances inducing them have an organic origin.

Regarding the limits of detection, there are some options to overcome this issue. First, doing an incubation period of 3–5 days guarantees that if the right conditions are met, the MNPs will be formed. In some suspensions from microbial origin, formation of MNPs can be immediate, but it can also take up to 3–5 days [11]. This is why it is important to follow reactions at different time-points for this period.

For the development of our proposed method, we can assume that even if there are no microbes currently present in the samples, but they were in contact with living organisms, then there will be extracellular or microbe-free compounds from those organisms in the samples. NPs formed from extracellular solutions are traditionally smaller than the intracellular ones [11]; therefore, these might be more difficult to detect. However, if the final suspension is concentrated by evaporation (as mentioned in step D, Section 2.1), then any MNPs will be in a higher amount in a smaller suspension volume, allowing for stronger optical density and easier color-detection.

The use of both AuNPs and AgNPs as indicators of life is possible because of their optical properties. They have a strong interaction with light, allowing for the typical bright color for which the localized surface Plasmon resonance is responsible [3]. It is actually this property that makes these NPs so suitable as potential biosignatures.

A potential biosignature is described by Des Marais et al. [57] as “a feature that is consistent with biological processes and that, when it is encountered, challenges the researcher to attribute it either to inanimate or to biological processes”. The AuNPs and AgNPs formation are here proposed as a method to screen for possible traces of life. A positive result, on its own, can be considered a potential biosignature, which is also referred to as an ambiguous biosignature by Chan et al. [48]. This type of biosignature leads to more research and data gathering in order to conclude whether there is presence or absence of life. Ambiguous signatures, in which the formation of AuNPs and AgNPs fit, are one of the main focuses of astrobiological studies [48]. Nevertheless, further investigation should still be done, by applying adequate characterization methods, confirming that any molecule/enzyme/protein responsible for the metal ions reduction has a biological source, and look for possible primary biomolecules or diagenetically altered biomolecules adhered to the NPs that might aid on the identification of any biogenic processes and exclude any false-positive results. For example, John et al. [58] did FTIR analysis on AgNPs, biosynthesized by *Pseudomonas* sp., and confirmed the presence of capping proteins on the NPs, which distinguished them from non-biogenic NPs.

Furthermore, special care should also be taken regarding sampling and sample handling and any necessary sample preservation. Possible contaminations should be accounted for, and standard measures should be used to avoid them.

## 3. Conclusions

The formation of MNPs has already found a wide range of applications and their synthesis is the focus of several research groups. Our proposal to use the biogenic formation of MNPs to screen samples to detect organic material and evidence of life is worth investigating further. The potential for applying this to extraterrestrial sample analysis is particularly promising for the astrobiological exploration of Mars, both as part of in situ testing in future space missions, as well as planned sample-return ones.

Our proposed approach is meant for preliminary screening of potential biosignatures, but might benefit from coupling with further downstream analysis of produced MNPs, for further insights into the source of their production (e.g., Raman spectroscopy). The extremely wide range of organic molecules capable of inducing biogenic MNPs increases the usefulness of this approach and might help to reduce some of the limitations of having to anchor it exclusively on our knowledge on terrestrial lifeforms.

## 4. Future Prospects

Any future extraterrestrial samples, analyzed in situ or retrieved and brought back to Earth from Mars or elsewhere, could follow the simple protocol proposed here in a lab-environment/facility. This sort of application would be a simple procedure to include in any post-sample-retrieval-space-mission design. It is not a costly step, and, due to its simplicity, it can be easily performed without the need for specialized equipment of personnel.

As a potential biosignature, this could also be used on Earth in locations often used as terrestrial analogs (e.g., Table 5). In many of these locations, life is frequently considered to be scarce or even non-existent, and testing our proposed method on samples from such locations could aid in confirming our hypothesis.

Additional studies are necessary on this topic, with different types of samples and from different provenances, to better understand and circumvent any possible issues. Better ways of detecting minimal MNPs formation are important to avoid any false-negative results. Analyzing any species screened as non-producers of MNPs would be relevant to confirm that they will actually form NPs under different conditions, or that they just produce undetectable amounts. Some biological examples of no production after such screenings that could be used in further studies, are the phloem of the plants’ species *Brassica juncea*, *Festuca rubra*, and *Medicago sativa* [24], stem and leaf extracts of *Calotropis procera* [60], the fungal strains *Alternaria solani* RCMB 07324 and *Aspergillus flavus* RCMB02426 [25], and bacterial strains isolated from soil polluted with industrial wastewater in Egypt [26].

More laboratorial assays on MNP stability and formation under mimicked space-conditions can contribute to fine-tune the method. Comparing the use of other metals for the formation of NPs by complex samples (e.g., microbial mixtures) can also be relevant to confirm the applicability we are here defending.

## Figures and Tables

**Table 1:** Nanotechnology-based methods and most commonly used MNPs for microbial detection.

MNPs Use for Specific Microbial Detection		References
**General Methods**		
Immunochromatographic strips	change color to indicate microbial detection	[9]
DNA labeled with MNPs	used as probes	[9]
Biobarcode amplification	uses two types of NPs, one for signal amplification (e.g., AuNPs) and another (e.g., magnetic NPs) for separation of nucleic acid sequences	[9]
Sensitivity enhanced immune-PCR	uses NPs conjugated with antibodies	[9]
Biosensors	have many applications and are classified according to: their bio-recognition element, e.g., enzyme-based, antibody-based (immunosensors), DNA hybridization-based (genosensors), aptamer-based (aptasensors) the transduction mechanism e.g., optical, electrochemical, piezoelectric, and thermal	[8,9,18]
**MNPs Types**		
Gold NPs (AuNPs)	used to detect bacteria (e.g., *Campylobacter jejuni*, *Escherichia coli*, *Lactobacillus* spp., *Salmonella typhimurium*, and *Staphylococcus aureus*) and bacterial spores, as fluorescent molecular beacons DNA molecular beacon ^1^ conjugates nanosensors (by being chelated with Tb^3+^ and Eu^3+^ or by being capped with polyethyleneimine, allowing colorimetric detection of bacteria) cysteine-modified NPs (with cysteine electrostatically adhered to their surface) aptamers (also for colorimetric detection of bacteria) antibody-functionalized NPs	[10,17]
Silver NPs (AgNPs)	preferred type of MNPs in many different nanotechnology formats for bacterial detection (*Escherichia coli*, and *Staphylococcus epidermidis*), e.g., protein-A-antibody-modified for detection with Raman spectroscopy lectin-sensitized anisotropic NPs citrate-capped NPs	[17]

^1^ Hairpin-shaped oligonucleotide probe, that has a fluorophore-quencher pair attached to its ends and becomes fluorescent upon hybridization to a target sequence [19].

**Table 2:** Biological producers of MNPs.

Producers		References
Secondary metabolites from organisms	Bioactive molecules: polysaccharides, enzymes, amino acids, vitamins, proteins, and organic acids.	[6]
	Amino acids have different side chains that can participate in sequestration and/or reduction of metal ions for NPs formation.	[20]
	Flavonoids, a large group of polyphenolic compounds, can actively chelate metal ions and reduce them to form NPs.	[6]
Egg-white proteins	Ovalbumin, ovotransferrin, and ovomucoid can act as reducing and capping agents to produce AgNPs.	[27]
Plants	Plant extracts can act as stabilizing or reducing agents and mediate reduction of metal salts to nanostructural elemental forms. They can contain biomolecules and organic compounds like alkaloids, flavonoids, saponins, steroids, and tannins, which can act as templating agents and be used successfully in the synthesis of several NPs. The NPs thus formed are capped by additional biomolecules present in the biological material.	[1,28,29]
	Agro-waste resources and food waste are seen as an eco-friendly method of synthesis and a sustainable way for effective utilization and management of plant wastes and biomass.	[28,29]
Algae	Green micro- and macro-algae, brown algae and, red algae (e.g., *Chlorella vulgaris*, *Cystophora moniliformis*, *Enteromorpha flexuosa*, *Gracilaria edulis*, *Klebsormidium flaccidium*, *Lemanea fluviatilis*, *Prasiola crispa*, *Sargassum wightii*, and *Stoechospermum marginatum*, among many others). Easy to grow in laboratories, have proteins in their membranes, and secrete secondary metabolites that can act as templating and stabilizing agents. Marine microalgae are constantly exposed to metal salts present in the water. Consequently, these are efficient in reducing metal salts into NPs and large-scale synthesis is cost-effective.	[29,30]
Fungi	Filamentous fungi are more efficient MNPs producers due to high tolerance to metals, easier handling, and capability to survive extreme conditions when in bioreactors. Most species are extremely efficient secretors of extracellular enzymes capable of reducing Ag^+^, facilitating scaled-up processes, downstream processing (biomass production and extraction). Many species described as capable of MNPs production; for example, AgNPs can be produced by *Aspergillus flavus*, *Aspergillus niger*, *Cladosporium cladosporioides*, *Epicoccum nigrum*, *Fusarium oxysporum*, *Fusarium solani*, *Penicillium fellutanum*, *Phanerochaete chrysosporium*, *Phoma glomerata*, and *Phoma macrostoma*.	[5,11,13]
	Yeasts (e.g., *Candida albicans*, *Candida glabrata*, *Magnusiomyces ingens*, *Pichia jadinii*, *Saccharomyces cerevisiae*, *Schizosaccharomyces pombe*, and *Yarrowia lipolytica*). Easy to use and manipulate in laboratories and good excretors of secondary metabolites that reduce and stabilize metal ions.	[31,32,33,34,35]
Bacteria	E.g., *Bacillus* spp., *Bacillus amyloliquefaciens* and *Bacillus subtillis*, and other bacteria from the genera *Acinetobacter*, *Escherichia*, *Lactobacillus*, and *Staphylococcus* have been used to produce AgNPs. Cyanobacteria, e.g., *Arthrospira platensis, Phormidium valderianum*, and *Plectonema boryanum*.	[22]
Archaea	Less studied than bacteria. E.g., haloarchaeal *Halococcus salifodinae* BK3 has been used to produce stable and spherical AgNPs in an intracellular synthesis process.	[36]
Viruses	Work as synthons or templates to produce MNPs. E.g., M13 bacteriophage, Chilo iridescent virus, Cowpea mosaic virus (CPMV) particles, and genetically engineered phage-based tobacco mosaic virus (TMV).	[20,37,38]

**Table 3:** Differences between chemical, physical, and biogenic synthesis of MNPs.

		Advantages	Disadvantages
Synthesis processes	Physical	Allow production of bulk amounts of NPs [21].	Higher production costs (energy and equipment), and higher NPs toxicity [12,21].
	Chemical	Cheaper for large-scale production [11]. Allow production of bulk amounts of NPs [21]. High purity and reproducible.	Less eco-friendly and higher health hazards (increased use of toxic chemicals, reducing agents, and non-polar solvents) [11,22]. Lengthier solvent removal processes [12].
	Biogenic	Simpler processes, easy to execute, clean, economical, and increased biocompatibility [13].	Generally lower biosynthesis efficiency and lengthier production time. Downstream processing of intracellular products is more complex and expensive [11].

**Table 4:** Different categories of biosignatures [47,48].

Biosignatures	
Substances	metabolic signatures, allotropes, elemental abundances, enantiomers, extracellular polymeric substances (EPS), isotopes, minerals, molecules, and their associated properties
Objects	morphological structures such as cells, concretions, fossils including trace-fossils and microbialites (stromatolites), and mats
Patterns	abundance of organic homologs and patterns of stable isotopic abundances between and within compounds

**Table 5:** Most relevant Mars analogs on Earth [59].

Environment Type:	Examples of Locations:
Acidic, iron-rich environments (similar to conditions believed to have existed on early Mars)	Rio Tinto, Spain low-temperature acidic springs, Yukon, Canada
Extremely dry locations	Atacama Desert, Chile (hyper-arid with presence of perchlorates) sand dunes and deserts (derived from wind-driven processes), Morocco hematite concretions (derived from water-driven processes), Utah canyonlands, USA
Locations with long-term exposure to extreme cold, extreme dryness, and radiation	Arctic and Antarctic sites, permafrost and ice surfaces in the Canadian High Arctic Siberia Svalbard Norway McMurdo dry valleys and Blood Falls
Volcanic and hydrothermal environments, with active and past volcanism and hydrothermal activity. Places with basalt, a common type of rock on Mars.	Iceland Hawaii (USA) Yellowstone National Park (USA) hydrothermal vents
